# Editorial: Evolutionary physiology

**DOI:** 10.3389/fphys.2022.992583

**Published:** 2022-08-25

**Authors:** Mercedes Okumura, Paulo Hilário Nascimento Saldiva, Mariana Matera Veras

**Affiliations:** ^1^ Laboratory for Human Evolutionary Studies, Department of Genetics and Evolutionary Biology, Institute of Biosciences, University of São Paulo, São Paulo, Brazil; ^2^ Departamento de Patalogia, Faculdade de Medicina da Universidade de São Paulo, São Paulo, Brazil; ^3^ Laboratório de Patologia Ambiental e Experimental LIM05, Hospital das Clínicas, Faculdade de Medicina, Universidade de São Paulo, São Paulo, Brazil

**Keywords:** evolutionary trends, urban environment, selective press, reproduction, temperature

It is possible to observe some evolutionary trends in our lineage since the origins of the first bipedal hominins about 7 million years ago. Since the origins of our species, Homo sapiens have been under intense selective pressures fostered by migratory movements and the colonization of new environments. During the Holocene, large human settlements influenced the evolution (adaptive and non-adaptive) of many species, including ours. Bipedalism favoured the development of the brain over a powerful body structure, promoting important changes in the mechanisms of reproduction, birth, food intake and absorption, as well as the control of body fluid homeostasis and temperature. Cities stimulated important changes in environmental, cultural, and social aspects of our lives. Such changes usually happen at a faster pace than evolutionary processes, a scenario that may promote an evolutionary mismatch, where proximate adaptive changes may result nowadays in the ultimate causes of disease.

As an illustrative example, the well-developed capacity to store sources of energy in fat tissue to overcome famine contributes to present-day obesity epidemics of highly processed food. Today, in addition to obesity, other health problems related to food are emerging, such as intolerance to gluten and lactose. After childhood, the lactase gene tends to be less expressed and may be associated with lactose intolerance. In this Research Topic, Wells et al. present their novel hypothesis that lactase persistence could have undergone rapid selection in different global regions under the unifying selective pressure of reducing maternal mortality risk associated with obstructed labour.

The rapid increase in global temperature may disturb the adaptive regional adaptation to weather achieved when our species moved from Africa to more extreme latitudes. Park et al. experimentally investigated how high temperatures (42°C) influence the levels of irisin, which is a myokine (protein secreted or released from skeletal muscle cells) and their results show that the concentration of irisin was related to oxidative stress and muscle damage, reinforcing the importance of temperature for our body homeostasis. Evidence of the impact of extreme temperature on health are increasing, and climate change will bring enormous challenges to our physiological adaptation to changing temperature.

Moreover, evolution is driven to maximize reproductive capacity, mostly in terms of biologic parameters. Is it true nowadays when human reproduction can be programmed in terms of time, professional or cultural aspects and mediated by medical interventions? The modern lifestyle seems to be working to decrease reproductive efficiency, among the main factors are: delayed childbearing, obesity, deficient nutrition, smoking, stressful life, and exposure to environmental pollutants. Two papers in this issue are related to modern techniques in assisted reproduction for infertility and investigation of chromosomal defects. Kong et al. investigated if the effectiveness and safety of a specific strategy for *in vitro* fertilization, the early cumulus cell removal. Besides all development in assisted reproductive technology, failure still occur, and later life effects of these technologies on the offspring’s health needs to be further investigated. Song et al. evaluated if the gender and age of the carrier (mother or father) of the chromosomal translocation, which is a common chromosome structural abnormality could impact blastocyst formation and pregnancy outcome. Presently, the propensity to survive and reproduce is also modulate by other aspects in a globalized world. Economic aspects and demographic aspects, large fluxes of people across the planet, and the emergence of new epizootic agents, promotes uneven possibilities to survive and reproduce to chronic and infectious diseases, as occurred in the COVID-19 pandemics.

Deans presents a thoughtful review regarding biological prescience and anticipation in organismal processes, which traditionally has been predominantly associated with the psychology of the human mind. The author makes a strong case for incorporating such a framework into the current Evolutionary Physiology paradigm in order to further understand such anticipatory responses as a unique type of biological processes.

The published articles in this Research Topic volume address a wide range of approaches within evolutionary physiology and reading them allows us to evaluate urban life from the perspective of the interaction of our physiology with the stimuli of the environment.

